# Mental health effects of infection containment strategies: quarantine and isolation—a systematic review and meta-analysis

**DOI:** 10.1007/s00406-020-01196-x

**Published:** 2020-10-06

**Authors:** Jonathan Henssler, Friederike Stock, Joris van Bohemen, Henrik Walter, Andreas Heinz, Lasse Brandt

**Affiliations:** 1 Department of Psychiatry and Psychotherapy, Charité Campus Mitte, Charité Universitätsmedizin Berlin, Corporate Member of Freie Universität Berlin, Humboldt-Universität Zu Berlin, and Berlin Institute of Health, Charitéplatz 1, 10117 Berlin, Germany; 2 Department of Psychiatry and Psychotherapy, University of Cologne Medical School, Cologne, Germany; 3 Berlin School of Mind and Brain, Berlin, Germany; 4 Bernstein Center of Computational Neuroscience Berlin, Berlin, Germany

**Keywords:** Isolation, Quarantine, Containment strategies, Psychological effects, Mental health, Mental disorder, Systematic review, Meta-analysis

## Abstract

Due to the ongoing COVID-19 pandemic, an unprecedented number of people worldwide is currently affected by quarantine or isolation. These measures have been suggested to negatively impact on mental health. We conducted the first systematic literature review and meta-analysis assessing the psychological effects in both quarantined and isolated persons compared to non-quarantined and non-isolated persons. PubMed, PsycINFO, and Embase databases were searched for studies until April 22, 2020 (Prospero Registration-No.: CRD42020180043). We followed PRISMA and MOOSE guidelines for data extraction and synthesis and the Newcastle–Ottawa Scale for assessing risk of bias of included studies. A random-effects model was implemented to pool effect sizes of included studies. The primary outcomes were depression, anxiety, and stress-related disorders. All other psychological parameters, such as anger, were reported as secondary outcomes. Out of 6807 screened articles, 25 studies were included in our analyses. Compared to controls, individuals experiencing isolation or quarantine were at increased risk for adverse mental health outcomes, particularly after containment duration of 1 week or longer. Effect sizes were summarized for depressive disorders (odds ratio 2.795; 95% CI 1.467–5.324), anxiety disorders (odds ratio 2.0; 95% CI 0.883–4.527), and stress-related disorders (odds ratio 2.742; 95% CI 1.496–5.027). Among secondary outcomes, elevated levels of anger were reported most consistently. There is compelling evidence for adverse mental health effects of isolation and quarantine, in particular depression, anxiety, stress-related disorders, and anger. Reported determinants can help identify populations at risk and our findings may serve as an evidence-base for prevention and management strategies.

**Electronic supplementary material:**

The online version of this article (10.1007/s00406-020-01196-x) contains supplementary material, which is available to authorized users.

## Introduction

Quarantine and isolation are main containment strategies intended to help protect the public by preventing the spread of contagious diseases. Both strategies primarily refer to a restriction of movement and limitation of personal contacts [1]. Quarantine, per definition, is used for persons that may have been exposed to the disease, while isolation is used for contagious persons that require separation from persons who are not infected. Findings from previous research pointed towards an increased risk for negative psychological outcomes, such as depression and anxiety, through isolation [2–4]. Quarantined persons may equally be at heightened risk for adverse mental health outcomes. A rapid review by Brooks et al. reported increased negative psychological outcomes including post-traumatic stress symptoms, confusion, and anger in persons under quarantine [5]. The authors concluded that important stressors were longer quarantine duration, infection fears, frustration, boredom, inadequate supplies, inadequate information, financial loss, and stigma [5]. Findings suggest that both containment strategies, quarantine and isolation, have negative impacts on psychological outcomes related to a broad spectrum of psychosocial stressors [2–5].

The need for investigation of mental health problems associated with containment strategies is further highlighted by the rising implementation of quarantine and isolation worldwide due to the currently ongoing COVID-19 pandemic. An unprecedented number of people worldwide is affected by quarantine or isolation [6]. The identification of individuals at elevated risk for adverse mental health effects seems mandatory. It has been suggested that vulnerable populations at risk for negative psychological outcomes before implementation of containment strategies, e.g. persons with mental illness, low income, or lack of social network, may be at particular greater risk during and after quarantine or isolation [4].

The World Health Organization (WHO) has included COVID-19 in the list of diseases and pathogens prioritized for research and development (R&D) in public health emergency contexts, which pose the greatest public health risk due to their epidemic potential, as insufficient countermeasures have been established [7]. Containment strategies are among the main countermeasures in this context [1] and systematic investigation of evidence concerning their psychological effects is urgently in need.

Single studies and reviews [4, 5] suggest an increased risk of negative psychological outcomes in persons under quarantine or isolation, but others presented partially contradicting results [8, 9]. Furthermore, prevalence estimates point towards elevated levels of adverse outcomes in quarantined or isolated populations [4], however, validity of these findings is often limited by the underlying uncontrolled study design. We, therefore, conducted a systematic literature review and meta-analysis of the mental health effects of quarantine and isolation, based on controlled primary study data. To the best of our knowledge, no meta-analysis including both quarantine and isolation exists to date.

## Methods

This is a systematic literature review and meta-analysis. The protocol of the project has been published on PROSPERO (Prospero Registration-No.: CRD42020180043). Methods followed guidelines by the Cochrane Collaboration for the conduction of systematic reviews [10].

### Search strategy

We searched PubMed, PsycINFO, and Embase databases for studies with no restrictions, from the beginning of the searched time period and until April 22, 2020, assessing the rate of psychological effects in quarantined/isolated persons compared to non-quarantined/non-isolated persons. Search entry is described in an online supplement (Supplement 1. Database search entry). Broad and specific search terms were combined to increase the likelihood of detecting eligible studies for our research aim. Among the specific search terms, we included a list of diseases and pathogens prioritized for research and development (R&D) in public health emergency contexts by the World Health Organization (WHO), such as COVID-19 [7].

Additional records were identified through manual searches of references of the included studies. We included no language restrictions and translations by a native speaker were acquired to test eligibility criteria of articles in languages other than English. Study authors were contacted in case of missing data. The search was carried out using Endnote X9.3 (Clarivate Analytics, Philadelphia, USA).

### Eligibility criteria

Trials were considered appropriate to test the hypothesis and included when they met the following criteria. First, observation of persons in quarantine or isolation was described. Second, quantitative assessment of psychological outcome parameters was performed. Third, comparators were persons not in quarantine or isolation. Fourth, data for the calculation of effect sizes and corresponding measures of dispersion were provided. Studies observing psychological outcome parameters by qualitative assessment only were excluded. Studies were excluded if they focused on specific subpopulations without primary infection control-association, such as isolated persons in prisons. Studies assessing correlations of mental health outcomes with varying durations of quarantine or isolation only were excluded from quantitative synthesis and reported in our qualitative synthesis of determinants.

### Selection of studies, data collection, and extraction

The entire literature search and study screening were carried out independently by two reviewers (FS, JVB). Consensus in unclear cases was reached via discussion with additional members of the reviewing team (LB, JH). Testing of eligibility criteria, study selection, and classification and coding of data into a predefined Excel spreadsheet (Microsoft Excel for Mac, Version 16.12, Microsoft Corporation, USA) followed recommendations by the Cochrane Collaboration Handbook [10] and were performed independently by two reviewers (LB, JH).

### Data extraction

Two reviewers (JH, LB) independently extracted data regarding characteristics of the study and study samples, as well as quantitative data on severity (mean scores) or frequency (incidence or prevalence) of mental health outcomes for each group or for the comparison between groups (e.g. relative risk, odds ratio), and the results of any determinant testing reported to reach statistical significance in the original studies. When multiple measures for the same outcome were reported, we extracted data in the following hierarchy: (1) continuous measures (mean scores), (2) categorical measures using the highest cut-offs defined by the authors of the original studies (i.e. the most severe manifestation of the disorder).

### Risk of bias

Risk of bias of studies was classified independently by two reviewers (LB, JH) according to the Newcastle–Ottawa Scale (NOS) [11] as recommended by the Cochrane Handbook [10] (Table 1). By summary assessment, all studies were classified as holding low or unknown/high risk of bias by taking into account bias from the three main domains selection, comparability, and exposure/outcome. Disagreements were resolved by consensus with additional review authors.

**Table 1:** Characteristics of included studies in the quantitative synthesis

Study	Country	Design	Participants	Containment cause	Containment procedure, mean duration	Outcome measures	Risk of bias
Bai et al. (2004) [25]	Taiwan	Cross-sectional	338 hospital staff	Contact with suspected SARS cases	Quarantine, 9d	Study-specific survey^a^	Unknown/high
Chua et al. (2004) [31]	Hong Kong	Cohort	224 patients	SARS-infection	Isolation, 1d	PSS	Low
Day et al. (2011) a [30]	US	Cohort	103 patients	MDR-infection	Isolation, 1d	HADS, depression or anxiety	Low
Day et al. (2011) b [43]	US	Cohort	36,112 patients	MDR-infection	Isolation, 9.5d (ICU: 17d)	ICD-9, depressive disorder, anxiety disorder	Low
Day et al. (2012) [44]	US	Cohort	45,266 patients	MDR-infection^b^	Isolation, n.s	ICD-9	Low
Day et al. (2013) [26]	US	Cohort	528 patients	MDR- or clostridium difficile-infection	Isolation, 1d/3d/7d	HADS, VAMS	Low
Findik et al. (2012) [18]	Turkey	Quasi-experimental	117 patients	Unspecified hospital infection	Isolation, 5d	HADS	Low
Gammon et al. (1998) [33]	UK	Quasi-experimental	40 patients	Unspecified infection	Isolation, 7d	HADS, Health Illness Scale, Self Esteem Scale	Low
Guilley-Lerondeau et al. (2017) [32]	France	Cohort	90 patients	Unspecified infection	Isolation, 3d	Spielberger scale	Low
Kennedy et al. (1997) [45]	UK	Cross-sectional	32 patients	MRSA-infection	Isolation, 14d	Functional independence measure, BDI, STAI, POMS	Unknown/high
Ko et al. (2006) [21]	Taiwan	Cross-sectional	1499 participants	SARS	Isolation, n.s	SARS questionnaire, Taiwanese Depression Questionnaire, Self- Perceived Health Questionnaire, Neighborhood Relationship Questionnaire	Unknown/High
Lau et al. (2016) [9]	Canada	Cohort	495 patients	Unspecified infection	Isolation, 6.2d	Charlson comorbidity score, clinical frailty, depression, anxiety, health-related quality of life, and patient satisfaction	Unknown/High
Lee et al. (2018) [27]	South Korea	Cohort	1800 hospital staff and 73 patients	MERS	Quarantine, 14d	IES-R, Mini International Neuropsychiatric Interview and HADS	Unknown/high
Liu et al. (2012) [24]	China	Cross-sectional	549 hospital staff	SARS	Quarantine, n.s	Center for Epidemiologic Studies Depression Scale, IES-R	Low
Lupion-Mendoza et al. (2015) [46]	Spain	Case–control	144 participants	Unspecified infection	Isolation, 5d	HADS, health care satisfaction	Low
Marjanovic et al. (2007) [28]	Canada	Cross-sectional	333 nurses	SARS	Quarantine, n.s	MBI-GS, STAXI, Schaufeli scale, SPOS, study-specific scales	Unknown/high
Mihashi et al. (2009) [19]	China	Cross-sectional	187 printing company workers, university faculty members and their families, and non-medicine students	SARS	Isolation, n.s	Psychological Disorder (> / = 7 GHQ)	Unknown/high
Soon et al. (2013) [22]	Singapore	Cross-sectional	40 patients	MDR-infection	Isolation, 6.8d	HADS, PSS	Low
Sprang and Silman (2013) [16]	US	Cross-sectional	398 parents	Unspecified infection	Quarantine or isolation, n.s	PTSD-RI, PCL-C	Unknown/high
Tarzi et al. (2001) [47]	UK	Cross-sectional	42 patients	MRSA-exposure	Isolation, 31.5d	Abbreviated Mental Test Score, Barthel Index, GDS, PDMS	Unknown/high
Taylor et al. (2008) [15]	Australia	Cross-sectional	2760 participants involved in horse industry	Equine influenza	Quarantine, several weeks	Kessler 10	Unknown/high
Wang et al. (2011) [8]	China	Cross-sectional	419 undergraduate students	H1N1	Quarantine, 7d	SRQ-20, IES-R	Low
Wassenberg et al. (2009) [29]	NL	Cross-sectional	126 patients	MDR-infection	Isolation, 1.5d	HADS, EQ-5D-VAS	Unknown/high
Wu et al. (2009) [17] Wu et al. (2008) [20]	China	Cross-sectional	549 hospital staff	SARS^c^	Quarantine, n.s	IES-R, study specific Alcohol Use Disorder symptoms, SARS questionnaire	Low

*d* day(s), *n.s.* not specified, *PSS* Perceived Stress Scale, *ICD-9* International Classification of Diseases, Ninth Revision, *HADS* Hospital Anxiety and Depression Scale, *VAMS* Visual Analog Mood Scale, *BDI* Beck depression Inventory, *STAI* State-Trait Anxiety Inventory, *POMS* Profile of Mood States, *SARS* severe acute respiratory syndrome, *MERS* Middle East Respiratory Syndrome, *MBI-GS* Maslach Burnout Inventory-General Survey, *STAXI* State-Trate Anger Expression Inventory, *SPOS* Survey of Perceived Organizational Support, *IES-R* Impact of Event Scale-Revised, *ICU* intensive care unit, *PTSD-RI* Post-Traumatic Stress Disorder reaction index, *PCL-*C PTSD CheckList-Civilian Version, *GDS* Geriatric Depression Scale, *PDMS* Peabody Developmental Motor Scales, *SRQ-20* 20-item Self Reporting Questionnaire, *EQ-5D-VAS* European Quality of Life 5 Dimensions 3 Level Version, *US* United States of America, *UK* United Kingdom, *CP* contact precautions

^a^SARS-related stress survey composed of acute stress disorder criteria according to the DSM-IV and related emotional and behavioural changes

^b^MDR-bacteria including MRSA, VRE and gram-negative bacteria

^c^Quarantining was defined, based on six questionnaire items, as quarantined as a result of being diagnosed with SARS or suspected of having SARS, or as having had direct contact with SARS patients either at work, at home, or in other places

### Data synthesis

We calculated standardized mean differences (SMD) and 95% confidence intervals (CIs) from outcome measures of the primary studies. If respective measures of dispersion were not available, we calculated CIs from *p* values as recommended in the Cochrane Handbook [10]. Stratified by our pre-defined mental health outcomes, effect sizes for comparisons between quarantined/isolated and non-quarantined/isolated groups were summarized using forest plots and tables. A quantitative synthesis of all these results was not possible due to the heterogeneity of the included studies in methodology, populations, and outcomes.

We, therefore, restricted quantitative syntheses to our pre-defined outcomes and to primary studies that provided data on categorical outcomes based on validated diagnostic criteria for mental disorders. From these, we calculated summary estimates (odds ratio and 95% CI) using random-effects models (DerSimonian and Laird method), as the studies differed in several methodological aspects. Effect sizes from different, non-overlapping subgroups of populations within a study were pooled using a fixed-effect model, as recommended in the Cochrane Handbook [10] (three-level meta-analytic approach). Heterogeneity among studies was quantified with the *I*^2^ statistic. Analyses were conducted according to the Cochrane Collaboration Handbook [10] and using Comprehensive Meta-Analysis V3 (Biostat, Engelwood, New Jersey).

Descriptive text was used to summarize the results of any determinant testing reported to reach statistical significance in the original studies.

## Results

**Fig. 1:**
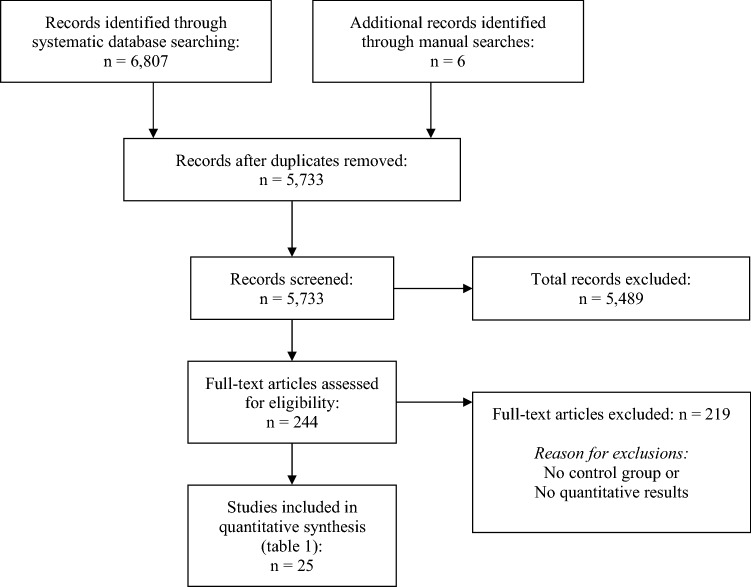
PRISMA flowchart

After screening of titles and abstracts of 6807 articles, 44 full-texts were assessed for eligibility. Of these, 25 studies, published between 1998 and 2018, were eligible for quantitative synthesis (Fig. 1). 16 studies observed isolation procedures, 8 studies observed quarantine procedures and one study observed quarantine and isolation procedures. Mean length of containment measures ranged from 1 to 31.5 days (Table 1). Three additional studies provided data on determinants only and were not included in quantitative synthesis [12–14].

### Main results

**Fig. 2:**
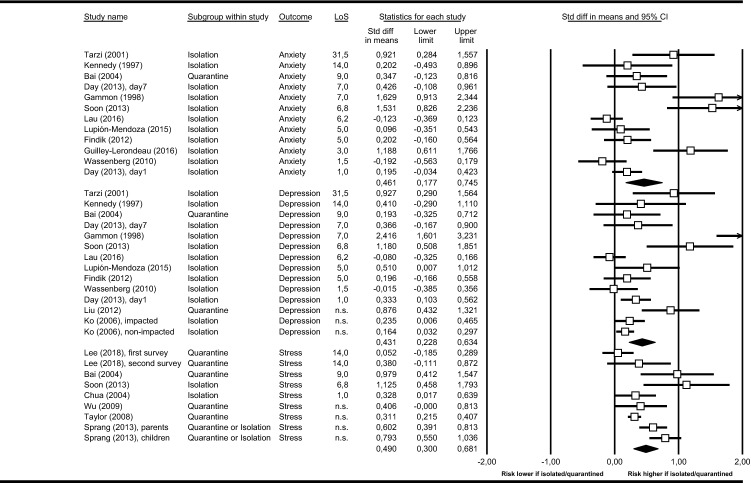
Primary outcomes. Outcomes stratified by anxiety, depression, and stress-related disorders as defined in the original study and summarized in standardized mean differences (SMD) and 95% CI. Summary estimates (black diamonds) are presented non-confirmatory and for estimate display only. LoS = length of stay in containment (i.e. duration of quarantine/isolation), stress = stress-related disorders

Pre-defined primary outcomes were depression, anxiety, and stress-related disorders. Figure 2 presents effect sizes from all studies providing data for these outcomes.

**Fig. 3:**
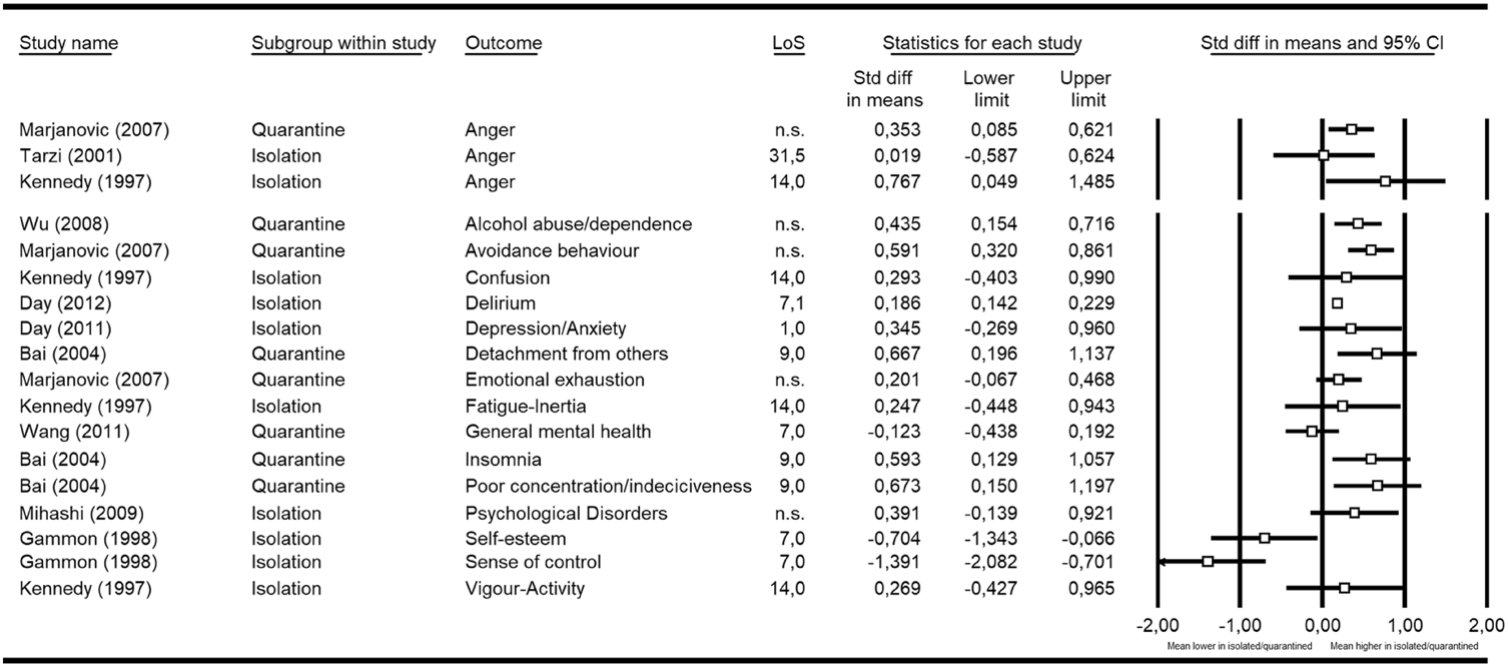
Secondary outcomes. Outcomes stratified as defined in the original study and summarized in standardized mean differences (SMD) and 95% CI. LoS = length of stay in containment (i.e. duration of quarantine/isolation)

Secondary outcomes were all other mental health outcomes, as presented in Fig. 3.

### Quantitative analysis of categorical outcomes

**Fig. 4:**
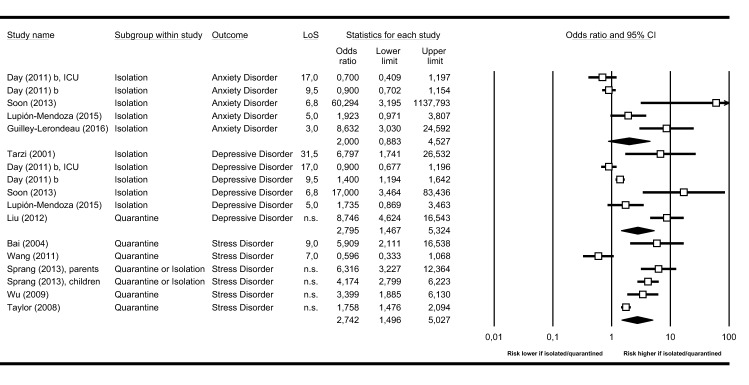
Categorical outcomes—quantitative synthesis. Categorical outcomes (i.e. disorders based on validated diagnostic criteria for mental disorders) stratified as defined in the original study and synthesized as odds ratio (OR) and 95% CI. LoS = length of stay (i.e. duration of quarantine/isolation). Anxiety disorder = anxiety based on validated diagnostic criteria for mental disorders. Depressive disorder = depression based on validated diagnostic criteria for mental disorders. Stress disorder = stress-related disorders based on validated diagnostic criteria for mental disorders

Quantitative synthesis of our pre-defined outcomes took into account primary study data on categorical outcomes based on validated diagnostic criteria for mental disorders (Fig. 4). Compared to non-quarantined/-isolated controls, individuals experiencing isolation or quarantine were at higher risk of depressive disorders (OR 2.795; 95% CI 1.467–5.324; *I*^2^: 91.1%), anxiety disorders (OR 2.0; 95% CI 0.883–4.527; *I*^2^: 86.5%), and stress-related disorders (OR 2.742; 95% CI 1.496–5.027; *I*^2^: 90.0%).

### Sensitivity analyses

Final ratings after assessment of methodological quality of included studies are summarized in Table 1. 14 out of 25 studies were considered to be of low risk of bias. Sensitivity analyses, restricted to studies of higher methodological rigor (i.e. low risk of bias), supported our main findings, i.e. observable higher risk of adverse mental health effects in the isolated/quarantined groups (Supplement 2).

### Subgroup analyses

An increase in all primary outcomes was observed in both quarantine and isolation. Both containment measures determined adverse mental health outcomes. Driven by the unequal number of available studies per group (i.e. quarantine or isolation), evidence-base is particularly strong for elevated levels of stress-related disorders in quarantined individuals and for depression and anxiety in isolated individuals (Fig. 2).

### Determinants of psychological effects

Determinants of psychological outcomes, reported to reach statistical significance in the primary studies, were: (results are from 1 study, if not otherwise specified).

#### Age

Younger age was associated with higher risk for stress-related disorders/PTSD (3 studies [15–17]), whereas persons > 55 years were at higher risk for depression [18].

#### Gender

Women were at higher risk for depression [18], PTSD [16], and general mental health impairments [8] (1 study each), while men were found to be at higher risk for (non-psychotic) psychological disorder of any kind [19] and at higher risk for alcohol use disorder [20] (1 study each).

#### Education

Lower levels of education were associated with more severe symptoms of stress-related disorders/PTSD [15] and higher risk of depression [18] (1 study each).

#### Income

Lower household income and financial loss or economic impact in pandemics was correlated with a higher risks for negative psychological effects, i.e. depression (2 studies [18, 21]), anxiety [13], anger [13], symptoms of stress-related disorders/PTSD [15], and unspecified psychological disorders [19] (1 study each). Lower income was also associated with higher persistence of symptoms of PTSD over 3 years [17]. Interestingly, higher household income was associated with higher risk of alcohol use disorder [20].

#### Social networks

Low levels of social capital, lower perceived social support, and lower neighborhood relationships were associated with higher levels of depression (2 studies [21, 22]) as well as anxiety, stress, and poor sleep quality (1 study [23]). Being single also determined higher levels of depression [24] and higher persistence of PTSD symptoms over 3 years [17] (1 study each). Health care workers (HCW) experienced higher levels of stigmatization [25]. One study reported higher levels of anger and anxiety with use of mail/texting and internet but not with telephone use in isolated, non-infected individuals [13].

#### History of mental illness

Previous mental illness and psychiatric inpatient admission was associated with greater anxiety (2 studies [13, 26]) and anger [13] levels. A history of trauma determined higher risk of depression [24]. Depression and PTSD symptoms and a history of alcohol use as a coping strategy were associated with a higher risk of consecutive alcohol use disorder [20].

#### Physical health status

Lower perceived current health status was associated with higher levels of depression [21].

#### Exposure to infection

Exposure to infected individuals (e.g., friends/relatives or patients for HCW) and higher perceived risk of infection were associated with higher rates of adverse mental health outcomes: risk of adverse mental health effects was highest with having been infected oneself [13, 19]. Health care workers (HCW) were at higher risk compared with administrative personnel and HCW were at higher risk the more intense they worked with infected patients. This association was reported for anxiety and anger [13], depression (2 studies [21, 24]), stress-related disorders/PTSD (3 studies [14, 17, 27]), emotional exhaustion (2 studies [25, 28]), insomnia [25], alcohol use disorder (AUD) [20], and any psychological disorders [19]. HCW with infection-related tasks were also reported to be at higher risk for persisting symptoms of PTSD one month after the end of infection containment measures [27]. Perception of the risk of health hazards due to infection was associated with a higher risk of symptoms of stress-related disorders/PTSD [8].

#### Satisfaction of patients and health care workers

For isolated/quarantined individuals, dissatisfaction with containment measures, supply, or the relationship to healthcare-personnel was associated with higher levels of anxiety and anger [13], stress-related disorders/PTSD (2 studies [8, 14]) and lower general mental health [8]. For HCW, lower trust in equipment and infection control initiatives determined higher levels of anger and emotional exhaustion, whereas higher organizational support was associated with lower anger and lower avoidance behavior [28].

#### Duration of quarantine/isolation

Increased length of quarantine or isolation positively correlated with higher levels of anger (2 studies [13, 28]), anxiety [13], avoidance behavior [28] and stress-related disorders/PTSD [14]. Independent of infection status, isolation was found to have negative psychological effects after 1 and particularly after 2 weeks [12]. Some studies [29, 30] did not find negative mental health effects in isolation of 1–3 days duration, whereas others [26, 31, 32] did.

#### Other

Altruistic acceptance of infection-risk was reported to be protective against depression [24] and stress-related disorders/PTSD [17]. Increased perceived stress was associated with higher levels of depression and anxiety [22]. Self-esteem and sense of control were inversely correlated with anxiety and depression [33]. Children of parents with symptoms of PTSD had themselves an elevated risk for PTSD [16].

## Discussion

This systematic review and meta-analysis yielded the following main results: Individuals experiencing quarantine or isolation are at heightened risk of depression, anxiety, stress-related disorders and anger compared to non-quarantined or non-isolated persons. Data for other mental health outcomes mainly resulted from single trials, but likewise strongly and coherently indicated increased adverse mental health effects in quarantined and isolated individuals.

The included studies were heterogeneous in methodology, definition of containment strategies, and outcome parameters. Determination of exact risk estimates is, therefore, limited and pooled effect size estimates should only serve as guiding values. In spite of this cautionary remark, our results provide compelling evidence for increased adverse mental health outcomes in isolated or quarantined individuals.

Sensitivity analyses, restricted to studies of higher methodological rigor, supported the main findings. Thus, even in light of the methodological diversity of the included studies, findings appear to be sufficiently robust to impact on and inform clinical decision-making. Since only 14 studies were considered “low” risk of bias, more studies of high methodological rigor are needed to determine precise risk estimates.

Our general findings are in line with previous research: Brooks et al. performed a rapid review of the literature including qualitative data and concluded that post-traumatic stress symptoms, confusion, and anger appear to be increased in persons under quarantine [5]. In the same vein, cases of suicide associated with quarantine were reported during an outbreak with severe acute respiratory syndrome (SARS) outbreak 2012–2013 [34]. Purssell et al. previously reported increased rates of anxiety and depression in hospital-isolated patients [2]. These findings confirm an increased risk of mental health problems for persons under quarantine or isolation.

To some extent, heterogeneity in observed effects from included studies may be attributable to different durations of quarantine or isolation. Some studies [29, 30] did not find negative mental health effects in isolation of 1–3 days duration, but others [26, 31, 32] did. After periods of 1 and particularly of 2 weeks, however, evidence for adverse mental health effects of isolation and quarantine becomes increasingly solid [12, 14, 28].

Our analyses of determinants overall indicated that persons with higher levels of psychosocial vulnerabilities and stressors appear to be at particular risk for negative psychological outcomes associated with quarantine and isolation. This is in agreement with previous findings, indicating that the association between stress and mental health problems is determined by a variety of psychological, behavioral, and biological determinants including psychosocial resources, patterns of coping, and comorbidities [35]. Our review suggests that lower levels of education [15, 18], low income and financial loss [13, 15, 18, 19, 21], and lack of social networks are important determinants of negative psychological outcomes including depression, anxiety, and stress-related disorders, partly persisting over years [17].

Histories of mental illnesses or previous traumas likewise were factors associated with an increased risk of adverse mental health outcomes, highlighting the importance of particular awareness towards the vulnerability of these individuals during quarantine or isolation. Importantly, studies that corrected for levels of psychological outcomes at baseline still detected increasing levels of negative psychological outcomes following with containment strategies [26, 27]. Even beyond that, however, persons with mental health disorders may experience increased difficulties in accessing mental health services, as well as day care centers and psychosocial networks, which are important for mental health outcomes. In line with previous studies [36] emphasizing the negative impact of social isolation and exclusion stress on mental disorders, containment procedures may, therefore, represent an independent risk factor for adverse mental health effects and are likely to affect larger parts of the general population. This independent risk factor, however, may particularly add up to pre-existing vulnerability.

We found cumulated evidence for elevated levels of anger in populations under quarantine or isolation, even increasing with ongoing duration of containment [13, 28]. This is of particular relevance during the current worldwide COVID-19 pandemic, as could be shown by concerns of increasing domestic violence and child abuse based on initial reports in populations affected by COVID-19 quarantine in Asia and Europe [37, 38].

A major important finding is the elevated risk of negative psychological effects for healthcare workers, particularly those with exposure to infected patients [8, 13, 14, 17, 19, 21, 24, 25, 27, 28]. Awareness has to be drawn to the finding [28] that their risk of negative psychological effects was determined by the perception of personal health hazards, organizational support, and trust in equipment, outlining the path for crucial prevention and management strategies to minimize adverse mental health effects for healthcare workers.

### Strengths and limitations

This review has several strengths and limitations. Strengths include the extensive database search and the duplication of screening, data extraction, and the thorough evaluation of the methodology and risk of bias of the studies. Also, by restricting eligibility of primary studies to those that used non-quarantined/-isolated populations as a comparator, we were able to calculate relative effect estimates with higher explanatory power.

However, this review also has several limitations. Studies reporting psychological outcomes only as secondary outcomes may not have been identified in the searches of electronic publication databases if these psychological outcomes were not reported in the title, abstract, keywords, or indexing terms. The use of the three large and relevant databases in this field and supplementary manual searches of all reference lists of included studies and related articles, however, should have minimized the risk of missing relevant studies.

Our meta-analysis confirmed the initial assumption that persons under quarantine or isolation are at risk for mental health problems. The representativeness and validity of our findings are, however, limited by the following aspects:

Limitations of the currently available evidence include (1) partial use of cross-sectional study designs, thus making temporality of events difficult to assess, (2) lack of power, and (3) frequent lack of consideration for important confounders, such as baseline mental health status.

The majority of included studies investigated single-person isolation measures. The scarcity of studies focusing specifically on quarantine in general population settings is a limitation of the current evidence and has to be accounted for when generalizing the findings of our meta-analysis. Additionally, during times of a pandemic, such as the current COVID-19 pandemic, populations may experience various degrees of restricted movement or limited personal contacts that do not necessarily coincide with systematically implemented quarantine or isolation. Clearly, conduction of adequately controlled studies is particularly challenging with regards to population-based quarantine measures. Our findings, however, are in accordance with and strengthened by results from additional uncontrolled studies [14, 39, 40], indicating that these differential containment strategies share indeed common adverse mental health effects. More research is needed to assess the differential effects of various degrees of movement restrictions and contact limitations on psychological outcomes in single person as well as population-based settings. Moreover, the studies in this meta-analysis are heterogenous with regard to study designs including definitions of the containment strategy, populations, and outcome parameters. Drawing conclusions from this meta-analysis to different subpopulations, such as children and geriatric subpopulations, and different procedures for implementing quarantine or isolation is, therefore, limited and should consider characteristics of the specific population and its specific reaction to a clearly defined containment strategy. Psychosocial factors relevant for the reaction to containment strategies and resulting mental health problems may significantly differ between subpopulations. To date, however, there is very limited specific evidence for each of the subpopulations only.

More controlled studies for specific subpopulations categorized according to mental and physical health, social support, and economic status are needed to further assess the generalizability of the findings. Generalizability would be further increased by implementation of standard diagnostic criteria of mental health problems, such as the Diagnostic and Statistical Manual of Mental Disorders (DSM) [41] or the International Statistical Classification of Diseases and Related Health Problems (ICD) [42].

### Implications

Persons under quarantine or isolation appear to be especially vulnerable for mental health problems associated with psychosocial adversities, such as social isolation, financial loss, inadequate supplies and information, stigma, and fear of infection [5].

This systematic review of the evidence identified a full range of adverse psychological effects in persons under quarantine or isolation. Further investigation should focus on the identification of moderating and protective factors and the development of effective prevention and management strategies aligned to populations of particular vulnerability.

Psychosocial challenges associated with containment strategies are of exceptional relevance due to the ongoing COVID-19 pandemic and the resulting frequent implementation of quarantine and isolation. Implementation of containment strategies should, thus, include consideration of increasing negative psychological outcomes associated with especially long durations of quarantine and isolation. Large groups of the general population may be affected, but individuals who are already facing psychosocial adversities before quarantine or isolation (including persons with low income, lack of social networks, or mental health problems) appear to be among those vulnerable groups at greatest risk for negative psychological outcomes. Health care workers showed a strong increase in negative psychological outcomes and stigma [14]. These effects might even be stronger in the ongoing COVD-19 pandemic taking into account that current measures of quarantine and in particular isolation are longer and affect large populations worldwide. Based on these findings, potential negative effects on mental health outcomes from infection containment strategies may possibly be reduced by several measures. Our findings highlight the need for organizational structures that can adapt to crisis management, sufficient equipment, and support for health care workers. Evidence strongly supports the inverse relationship between trust in equipment or organizational support and adverse mental health effects in this population at particular high risk for negative psychological outcomes. For persons with mental health disorders, maintenance of access to mental health care services should be of high priority. Targeted mental health prevention and intervention strategies for these populations at risk are urgently needed [5]. Moreover, the findings of this meta-analysis support the implementation of recently recommended measures to mitigate the potential negative psychological effects of quarantine, such as keeping the duration of the containment as short as possible, but as long as needed, providing adequate supplies for basic needs for quarantined households, providing persons with as much information as possible regarding the reason for the quarantine, and effective and rapid communication [5].

## Conclusion

Persons under quarantine or isolation are at heightened risk of mental health problems, in particular depression, anxiety, stress-related disorders and anger. Experiencing quarantine or isolation was found to represent an independent risk factor for adverse mental health outcomes. These findings highlight the need for mental health prevention strategies for populations at risk, particularly health care workers exposed to infection and individuals who already were facing psychosocial adversities before quarantine or isolation including those with low income, lack of social networks, or mental health problems.

## Electronic supplementary material

Below is the link to the electronic supplementary material.

Supplementary file1 (DOCX 17 kb)

Supplementary file2 (DOCX 17 kb)
