# Supplementary material for: Mental health effects of infection containment strategies: quarantine and isolation—a systematic review and meta-analysis
Source: Eur Arch Psychiatry Clin Neurosci. 2020 Oct 6;271(2):223–34. doi: 10.1007/s00406-020-01196-x (PMC7538183; doi:10.1007/s00406-020-01196-x)
Supplement: Supplementary file 1 — Supplementary file1 (DOCX 17 kb) [file 406_2020_1196_MOESM1_ESM.docx]

**Supplement 1**

**Figure 1: *Database search entry***

*Search term for the systematic literature search as Pubmed:*

((((((infection[Text Word] OR infected[Text Word] OR infective[Text Word] OR infectious[Text Word] OR communicable[Text Word] OR covid[Text Word] OR covid-19[Text Word] OR ncov[Text Word] OR coronavirus[Text Word] OR mers[Text Word] OR sars[Text Word] OR outbreak[Text Word] OR epidemic[Text Word] OR pandemic[Text Word] OR crimean-congo haemorrhagic[Text Word] OR ebola[Text Word] OR marburg virus[Text Word] OR lassa[Text Word] OR nipah[Text Word] OR henipaviral[Text Word] OR rift valley[Text Word] OR zika[Text Word] OR tuberculosis[Text Word]))) OR "Disease Outbreaks"[Mesh]))

AND

((((quarantine*[Text Word] OR isolation[Text Word] OR isolated[Text Word]))) OR ((("Patient Isolation"[Mesh]) OR "Quarantine"[Mesh]))))

AND

((((((((((("Mental Disorders"[Mesh]) OR "Mental Health"[Mesh]) OR "Anger"[Mesh]) OR "Hostility"[Mesh]) OR "Suicide"[Mesh]) OR "Behavioral Symptoms"[Mesh])) OR (((("Adaptation, Psychological"[Mesh] OR "Stress, Psychological"[Mesh]) OR "Delirium"[Mesh]) OR "Anxiety"[Mesh]) OR "Emotions"[Mesh]) OR "Violence"[Mesh])) OR "Resilience, Psychological"[Mesh]))

*Search term for the systematic literature search as Embase:*

| 1. exp violence/ |  |
| --- | --- |
| 2. exp psychological resilience/ |  |
| 3. exp epidemic/ |  |
| 4. (infection or infected or infective or infectious or communicable or covid or covid-19 or ncov or coronavirus or mers or sars or outbreak or epidemic or pandemic or crimean-congo haemorrhagic or ebola or marburg virus or lassa or nipah or henipaviral or rift valley or zika or tuberculosis).ab. or (infection or infected or infective or infectious or communicable or covid or covid-19 or ncov or coronavirus or mers or sars or outbreak or epidemic or pandemic or crimean-congo haemorrhagic or ebola or marburg virus or lassa or nipah or henipaviral or rift valley or zika or tuberculosis).ti. or (infection or infected or infective or infectious or communicable or covid or covid-19 or ncov or coronavirus or mers or sars or outbreak or epidemic or pandemic or crimean-congo haemorrhagic or ebola or marburg virus or lassa or nipah or henipaviral or rift valley or zika or tuberculosis).kw. |  |
| 5. exp isolation/ |  |
| 6. isolation.ab. or isolation.ti. or isolation.kw. |  |
| 7. quarantine.ab. or quarantine.ti. or quarantine.kw. |  |
| 8. patient isolation.ab. or patient isolation.ti. or patient isolation.kw. |  |
| 9. exp quarantine/ |  |
| 10. exp patient isolation/ |  |
| 11. exp mental disease/ |  |
| 12. exp mental health/ |  |
| 13. exp depression/ |  |
| 14. exp anger/ |  |
| 15. exp hostility/ |  |
| 16. exp suicide/ |  |
| 17. exp posttraumatic stress disorder/ |  |
| 18. exp delirium/ |  |
| 19. exp self esteem/ |  |
| 20. exp aggression/ |  |
| 21. exp stress/ |  |
| 22. (mental disorders or mental disorder or mental health or mental illness or suicide or suicidal or anger or hostility or aggression or depression or depressive or depressed or anxiety or PTSD or stress or distress or self-esteem or delirium or delir or anxiousness or psychological disorder or psychological disorders or psychological well-being or psychological effects or emotion).ab. or (mental disorders or mental disorder or mental health or mental illness or suicide or suicidal or anger or hostility or aggression or depression or depressive or depressed or anxiety or PTSD or stress or distress or self-esteem or delirium or delir or anxiousness or psychological disorder or psychological disorders or psychological well-being or psychological effects or emotion).ti. or (mental disorders or mental disorder or mental health or mental illness or suicide or suicidal or anger or hostility or aggression or depression or depressive or depressed or anxiety or PTSD or stress or distress or self-esteem or delirium or delir or anxiousness or psychological disorder or psychological disorders or psychological well-being or psychological effects or emotion).kw. |  |
| 23. 5 or 6 or 7 or 8 or 9 or 10 |  |
| 24. exp anxiety/ |  |
| 25. 11 or 12 or 13 or 14 or 15 or 16 or 17 or 18 or 19 or 20 or 21 or 24 |  |
| 26. 3 or 4 |  |
| 27. 1 or 2 or 25 |  |
| 28. 22 or 27 |  |
| 29. (violence or resilience).ab. or (violence or resilience).ti. or (violence or resilience).kw. |  |
| 30. 28 or 29 |  |
| 31. 23 and 26 and 30 |  |
| 32. 7 or 9 |  |
| 33. 30 and 32 |  |
| 34. 31 or 33 (final search entry used)  *Search term for the systematic literature search as PsycINFO(Ebscohost):*   \| \| **#** \| **Query** \| \| --- \| --- \| \| S19 \| S5 OR S18 \| \| S18 \| S14 OR S16 \| \| S17 \| (S1 AND S6 AND S7) AND (S14 OR S16) \| \| S16 \| S1 AND S6 AND S7 \| \| S15 \| (S7 AND S12) AND (S1 AND S6 AND S7) \| \| S14 \| S7 AND S12 \| \| S13 \| S7 AND S10 \| \| S12 \| S9 OR S10 \| \| S11 \| (MA quarantine) AND (S7 AND S10) \| \| S10 \| MA quarantine \| \| S9 \| TI quarantine OR AB quarantine OR KW quarantine \| \| S8 \| ( S1 AND S6 AND S7 ) OR S5 \| \| S7 \| S2 OR S3 \| \| S6 \| S4 OR S5 \| \| S5 \| MA quarantine OR MA patient isolation \| \| S4 \| TI ( Quarantine OR quarantined OR isolation OR isolated ) OR AB ( Quarantine OR quarantined OR isolation OR isolated ) OR KW ( Quarantine OR quarantined OR isolation OR isolated ) \| \| S3 \| MA mental disorders OR MA mental health OR MA mental illness OR MA suicide OR MA anger OR MA ( hostility or aggression ) OR MA depression OR MA anxiety OR MA stress OR MA delirium OR MA anxiousness OR MA ( ptsd or post traumatic stress disorder ) OR MA self-esteem OR MA psychological effects OR MA psychological disorders OR MA psychological well-being OR MA Resilience OR MA violence \| \| S2 \| TI ( mental disorders OR mental disorder OR mental health OR mental illness OR suicide OR suicidal OR anger OR hostility or aggression OR depression OR depressive OR depressed OR anxiety OR PTSD OR stress OR distress OR self esteem OR delirium OR delir OR anxious* OR psychological disorder OR psychological disorders OR psychological well-being OR psychological effects OR resilience OR violence ) OR AB ( mental disorders OR mental disorder OR mental health OR mental illness OR suicide OR suicidal OR anger OR hostility or aggression OR depression OR depressive OR depressed OR anxiety OR PTSD OR stress OR distress OR self esteem OR delirium OR delir OR anxious* OR psychological disorder OR psychological disorders OR psychological well-being OR psychological effects OR resilience OR violence ) OR KW ( mental disorders OR mental disorder OR mental health OR mental illness OR suicide OR suicidal OR anger OR hostility or aggression OR depression OR depressive OR depressed OR anxiety OR PTSD OR stress OR distress OR self esteem OR delirium OR delir OR anxious* OR psychological disorder OR psychological disorders OR psychological well-being OR psychological effects OR resilience OR violence ) \| \| S1 \| (TI ( infection OR infected OR infective OR infectious OR communicable OR covid OR covid-19 OR ncov OR coronavirus OR mers OR sars OR outbreak OR epidemic OR pandemic OR crimean-congo haemorrhagic OR ebola OR marburg virus OR lassa OR nipah OR henipaviral OR rift valley OR zika OR tuberculosis ) OR AB ( infection OR infected OR infective OR infectious OR communicable OR covid OR covid-19 OR ncov OR coronavirus OR mers OR sars OR outbreak OR epidemic OR pandemic OR crimean-cong haemorrhagic OR ebola OR marburg virus OR lassa OR nipah OR henipaviral OR rift valley OR zika OR tuberculosis ) OR KW ( infection OR infected OR infective OR infectious OR communicable OR covid OR covid-19 OR ncov OR coronavirus OR mers OR sars OR outbreak OR epidemic OR pandemic OR crimean-cong haemorrhagic OR ebola OR marburg virus OR lassa OR nipah OR henipaviral OR rift valley OR zika OR tuberculosis )) OR ( MA epidemic OR MA pandemic OR MA outbreak ) \| \| \| --- \| --- \| --- \| --- \| --- \| --- \| --- \| --- \| --- \| --- \| --- \| --- \| --- \| --- \| --- \| --- \| --- \| --- \| --- \| --- \| --- \| --- \| --- \| --- \| --- \| --- \| --- \| --- \| --- \| --- \| --- \| --- \| --- \| --- \| --- \| --- \| --- \| --- \| --- \| --- \| --- \| |  |
