# Supplementary material for: Mental health effects of infection containment strategies: quarantine and isolation—a systematic review and meta-analysis
Source: Eur Arch Psychiatry Clin Neurosci. 2020 Oct 6;271(2):223–34. doi: 10.1007/s00406-020-01196-x (PMC7538183; doi:10.1007/s00406-020-01196-x)
Supplement: Supplementary file 2 — Supplementary file2 (DOCX 17 kb) [file 406_2020_1196_MOESM2_ESM.docx]

**Supplement 2**

Forest Plot: Main outcomes – Sensitivity Analysis: Studies of higher methodological quality only (low risk of bias)

Outcomes stratified by symptoms anxiety, depression, and stress-related disorders as defined in the original study and summarized in standardized mean differences (SMD) and 95% CI. Summary estimates (black diamonds) are presented non-confirmatory and for estimate display only. LoS = Length of Stay in containment (i.e. duration of quarantine/isolation), Stress = stress-related disorders.
